# Effectiveness of Membrane Filtration to Improve Drinking Water: A Quasi-Experimental Study from Rural Southern India

**DOI:** 10.4269/ajtmh.15-0675

**Published:** 2016-11-02

**Authors:** Mark Rohit Francis, Rajiv Sarkar, Sheela Roy, Shabbar Jaffar, Venkata Raghava Mohan, Gagandeep Kang, Vinohar Balraj

**Affiliations:** 1 Division of Gastrointestinal Sciences, Christian Medical College, Vellore, India; 2 London School of Hygiene and Tropical Medicine, London, United Kingdom; 3 Department of Community Health, Christian Medical College, Vellore, India; 4 Society for Applied Studies, Vellore, India

## Abstract

Since point-of-use methods of water filtration have shown limited acceptance in Vellore, southern India, this study evaluated the effectiveness of decentralized membrane filtration 1) with safe storage, 2) without safe storage, versus 3) no intervention, consisting of central chlorination as per government guidelines, in improving the microbiological quality of drinking water and preventing childhood diarrhea. Periodic testing of water sources, pre-/postfiltration samples, and household water, and a biweekly follow up of children less than 2 years of age was done for 1 year. The membrane filters achieved a log reduction of 0.86 (0.69–1.06), 1.14 (0.99–1.30), and 0.79 (0.67–0.94) for total coliforms, fecal coliforms, and *Escherichia coli*, respectively, in field conditions. A 24% (incidence rate ratio, IRR [95% confidence interval, CI] = 0.76 [0.51–1.13]; *P* = 0.178) reduction in diarrheal incidence in the intervention village with safe storage and a 14% (IRR [95% CI] = 1.14 [0.75–1.77]; *P* = 0.530) increase in incidence for the intervention village without safe storage versus no intervention village was observed, although not statistically significant. Microbiologically, the membrane filters decreased fecal contamination; however, provision of decentralized membrane-filtered water with or without safe storage was not protective against childhood diarrhea.

## Introduction

Worldwide, an estimated 1.1 billion people do not have access to a safe source of drinking water.^1^ Water-borne pathogens of fecal origin are known to be associated with nearly 751,000 diarrheal deaths in children less than 5 years of age, more than half of which occur within the first year of life.^2^ In India alone, an estimated 212,000 children under 5 years of age died due to diarrheal diseases in 2010, making India a leading contributor to the global burden.^2^,^3^ Better access to and use of safe drinking water is crucial in preventing child deaths from diarrhea in low- and middle-income countries.^4^

In rural southern India, ground water is pumped from deep borewells into overhead tanks and distributed through subterranean or surface-level water pipelines to communities at least once a day.^5^–^8^ Despite a piped drinking water supply in most southern Indian villages, the quality of drinking water is poor.^9^,^10^ In rural and urban Vellore, multiple studies have demonstrated fecal contamination of drinking water, likely due to poor design and maintenance of water supply systems, inadequate water treatment, and prolonged household storage.^7^,^10^–^12^ Additional chlorination and solar disinfection have shown efficacy in reducing fecal contamination of drinking water at the point of use; however, poor uptake has led to only limited health gains from these interventions.^11^,^13^

Decentralized water treatment solutions provide an important alternative to traditional source-based and point-of-use water treatment.^14^,^15^ Small-scale systems are decentralized solutions that cater to several families or a small community and, by definition, are smaller than centralized systems.^15^ Small-scale systems such as the water treatment and refill kiosks in Indonesia, India, Bangladesh, Ghana, Nigeria, and other developing countries have been used to provide microbiologically safe drinking water to urban residents.^14^,^15^ A recent study from Indonesia reported reduced diarrhea among children from families using water kiosks.^14^

Membrane filtration systems have long been used for water and waste-water treatment, with applications primarily in reverse osmosis plants in water-scarce regions.^15^,^16^ Of the membrane filters, ultrafiltration membranes with a typical pore size between 0.002 and 0.1 μm have shown higher removal of pathogens such as *Cryptosporidium*, *Giardia*, and bacteria, viruses, and parasites.^15^

For this report, we evaluated a source-based, low-pressure membrane filtration system which operates without electricity or conditioning materials such as glycerol or ethanol, and has been used in emergency and disaster relief situations such as after the tsunami of 2004 in Sri Lanka, India, and Indonesia, and other humanitarian installations in 16 other low-income countries.^17^,^18^ In a preliminary study, the membrane filtration system effectively decontaminated drinking water in a residential campus in Vellore, over a 6-month period.^19^ Herein, we report the results of a community-based interventional study which evaluated the effectiveness of a commercially available membrane filter, the Skyhydrant^™^ water filtration system with a safe storage container versus without a safe storage container and central chlorination as per government guidelines in improving the microbiological quality of drinking water and preventing childhood diarrhea in rural southern India.

## Materials and Methods

### Study design and sample size.

The study was a three-arm (one village per arm), nonrandomized interventional trial.

1) Village 1: Households in the village received drinking water filtered by the membrane filtration system, that is, intervention-filtered water.
2) Village 2: Households in the village received both intervention-filtered water and a safe storage container with a narrow neck and a tap.
3) Village 3: The village was asked to continue water treatment, as per the existing guidelines, of adding 0.5 ppm bleaching powder to overhead tanks once fortnightly, and to collect water as per their normal practice.

The sample size calculation was based on an anticipated 25% reduction in under-two diarrheal incidence in the intervention village without safe storage versus no intervention and assuming three episodes of diarrhea per child per year^20^ in the no intervention village. Adding a 10% dropout rate to the estimates resulted in a sample size of 80 children per village, that is, a total of 240 children to be followed up for a period of 1 year.

### Study setting and participant eligibility.

**Figure 1.**
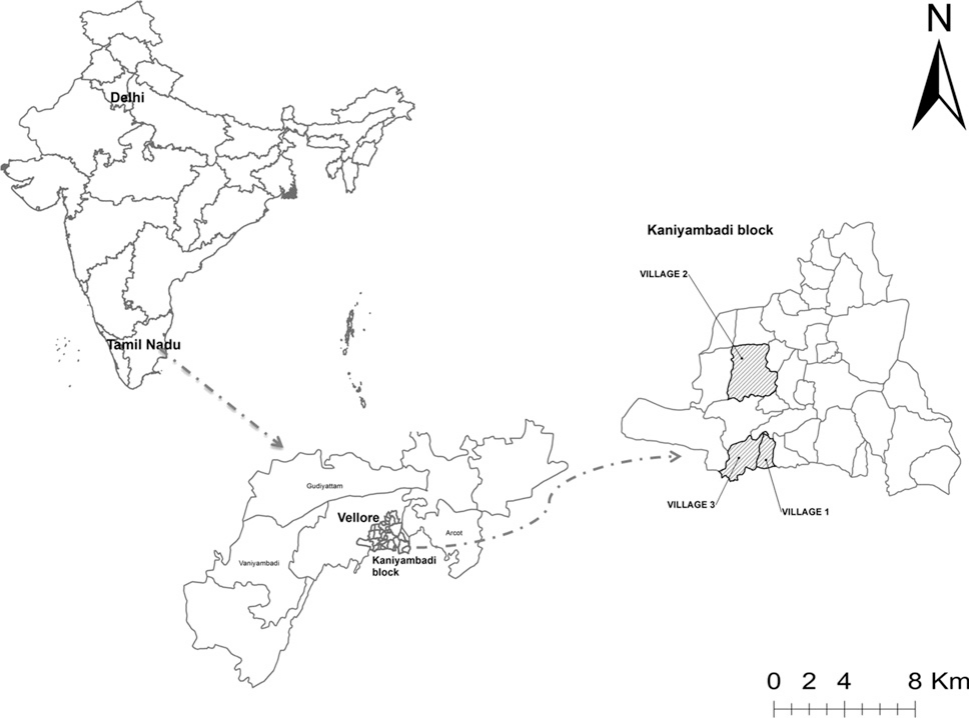
Map of the study villages.

The study was conducted between October 2013 and October 2014 in three large villages in the Kaniyambadi block (a rural administrative unit) of Vellore, Tamil Nadu, in southern India (Figure 1

). The Kaniyambadi block, comprising 85 villages (population = 104,792) is a demographic surveillance site of the Community Health Department (CHAD) of the Christian Medical College (CMC), Vellore. A list of villages with at least 80 children under 2 years of age or with expected births resulting in 80 children under 2 years of age were obtained from the CHAD census data, and three villages, Sholavaram, Kilarasampet, and Nanjukondapuram were selected as they were easily accessible by road, at a suitable distance from each other (between 1 and 5 km), and were representative of the larger villages in the block with respect to socioeconomic constitution, primary occupation, water sourcing, quality, and child-rearing practices.

The primary livelihood of the heads of household in the villages was farming or farm work, skilled work (masons, electricians, painters, and drivers) and unskilled work (street vendors or manual laborers). Women in the village were mostly housewives with a few intermittently employed during harvest season and school-aged children studied in government-run primary schools. The villages are mainly dependent on piped drinking water from their own deep borewells and pumped through a subterranean distribution network to taps on each street. Water supply is intermittent and lasts for 1–3 hours each day. Families collect drinking water in plastic or metal pots and store the pots at home for further use.

This study was carried out after obtaining clearance from the Institutional Review Board of CMC, Vellore. Verbal consent was obtained from the participating community through village meetings held to explain the nature, scope, and duration of the study. Intervention allocation was purposive, and the two villages with the most cooperative leaders, Sholavaram and Kilarasampet, were considered as intervention villages. A list of all households with recent births and children under 2 years of age was obtained from existing CHAD census databases and all households were sequentially approached for participation based on the obtained list. Households were enrolled after obtaining written informed consent. Children under 2 years of age in the enrolled households were followed up for a period of 1 year, or until their second birthday, or until the end of the study. The field staff kept track of antenatal women expected to deliver during the study period and continued to recruit children until August 2014.

### Intervention.

#### Membrane filtration system.

Households in Villages 1 and 2 were provided filtered water from a membrane filtration system. The membrane filtration system used was the Skyhydrant^™^ water filtration system (Skyjuice^™^ Foundation Inc., New South Wales, Australia), a commercially available gravity-fed, source-based water filtration system with a high throughput costing around INR 150,000 or USD 2,300. It uses a series of hollow fiber membrane tubes (about 1 m in length) composed of polyvinylidine fluoride with a pore size of 0.04 μm to filter water. Raw water passes in through a vent and out through another vent for collection or storage. Cleaning handles can be rotated to clean the internal filter module manually. The recommended head pressure for raw water flow of 3–6 psi, is achieved by positioning the reservoir tanks on the roof at a height of 3 m, which produces between 500 and 700 L of potable drinking water per hour.^20^ Additional detail on membrane filter installation and maintenance are available in another manuscript.^22^

#### Safe storage container.

Households in Village 2 were provided a polyvinylchloride container (25-L capacity, cost: INR 260 or USD 5) with a lid, tap, and a handle, for storage of filtered water. Study households were encouraged to rinse their containers with the filtered water before every collection. Single intervention and control households were asked to collect and store water as per their normal practice.

### Outcome assessment.

#### Water quality.

Village leaders helped contract existing pump drivers, responsible for daily distribution of water, for the maintenance and use of the Skyhydrant in each study village. Water samples were collected every month on a rotating schedule from the overhead tanks in each village (primary water sources) and pre- and postfiltration samples from each membrane filtration unit. Samples were also collected from 10% of the study households from the in-use drinking container once every 2 months. All water samples were collected in sterile, 250-mL polypropylene bottles with a stopper. Prefiltration samples were collected from a water tap connected to the pipe feeding water from the raw water storage tank to the membrane filtration system and postfiltration samples were drawn from the postfiltration storage tanks. The field workers were trained to collect and transport the samples per protocol.^11^,^23^ Taps were flamed for 2 minutes and water was allowed to flow for an additional minute before collection. Household container and source (overhead tank) samples were drawn using a designated scoop with a handle which was washed with distilled water and dried before and after every sample collection. The samples were tested for pH, nitrates, hardness, residual chlorine, and total dissolved solids (TDS) using standard testing kits (HiMedia Laboratories Pvt. Ltd., Mumbai, India). In addition, total coliforms (TC) colony-forming unit (CFU)/100 mL, fecal coliforms (FC) CFU/100 mL, and *Escherichia coli* CFU/100 mL were enumerated using MacConkey and M-FC media (HiMedia Laboratories Pvt. Ltd.). The range of detection for bacteria was 0–300 CFU/100 mL.

#### Diarrheal surveillance.

Trained field workers (one per village) visited households once during the first 4 days of the week and telephoned in the subsequent 3 days to achieve twice-weekly surveillance of each study household. They obtained information from the mother or the primary caregiver and recorded episodes of diarrhea in children less than 2 years of age in the preceding days. Diarrhea was defined as “passage of three or more loose or watery stools in 24 hours or, in case of infants, more frequent passage than normal.”^24^ Episodes of diarrhea were distinguished if they occurred at least 48 hours after cessation of the previous episode.^24^

### Data collection, entry, and analysis.

A baseline questionnaire administered in October 2013 (and subsequently for new entrants) captured demographic details, socioeconomic characteristics, water usage, water handling, and hygiene practices. Surveillance data were double entered using EpiInfo 2002 (Centers for Disease Control and Prevention, Atlanta, GA) software. Laboratory water sampling reports, maintenance logs, and migration details were compiled in Microsoft Excel 2010 spreadsheets (One Microsoft Way, Redmond, WA). Data analysis was performed using STATA for Windows version 12.0 (StataCorp, College Station, TX).

Baseline comparisons for selected demographic, socioeconomic, water usage, and hygiene variables were performed using χ^2^ test or Fisher's exact test for categorical variables and the analysis of variance F-test (with Scheffé's test for pairwise comparisons between villages) for continuous variables.

Arithmetic means of the microbiological parameters were presented using a Poisson distribution for the microbiological parameters and compared for the source and household water samples by study village and paired membrane filter pre- and postfiltration samples. The effectiveness of the membrane filters in reducing microbiological counts was presented as log-reduction values (LRVs) calculated as:

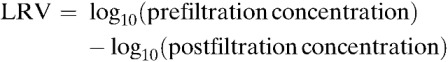


The LRVs are only presented for samples where the prefiltration microbiological concentration was > 0 CFU/100 mL sampled water.

The primary outcome, diarrheal incidence in children under 2 years of age was analyzed using an intention-to-treat analysis. Univariate survival analysis was performed for each of the baseline variables regressed against the incidence of diarrhea in children of the study. Type of house, socioeconomic status, and agent used for hand washing were found to be associated with childhood diarrhea at *P* < 0.10. These variables were adjusted using Poisson survival regression models and were presented as diarrheal incidence rate ratios (IRR [95% confidence interval, CI]) with a shared frailty to account for clustering of children within study households. We also investigated the effect of the microbiological quality of household drinking water on diarrheal incidence in the subset of intervention households (*N* = 122) whose samples had been collected.

## Results

### Participants.

**Figure 2.**
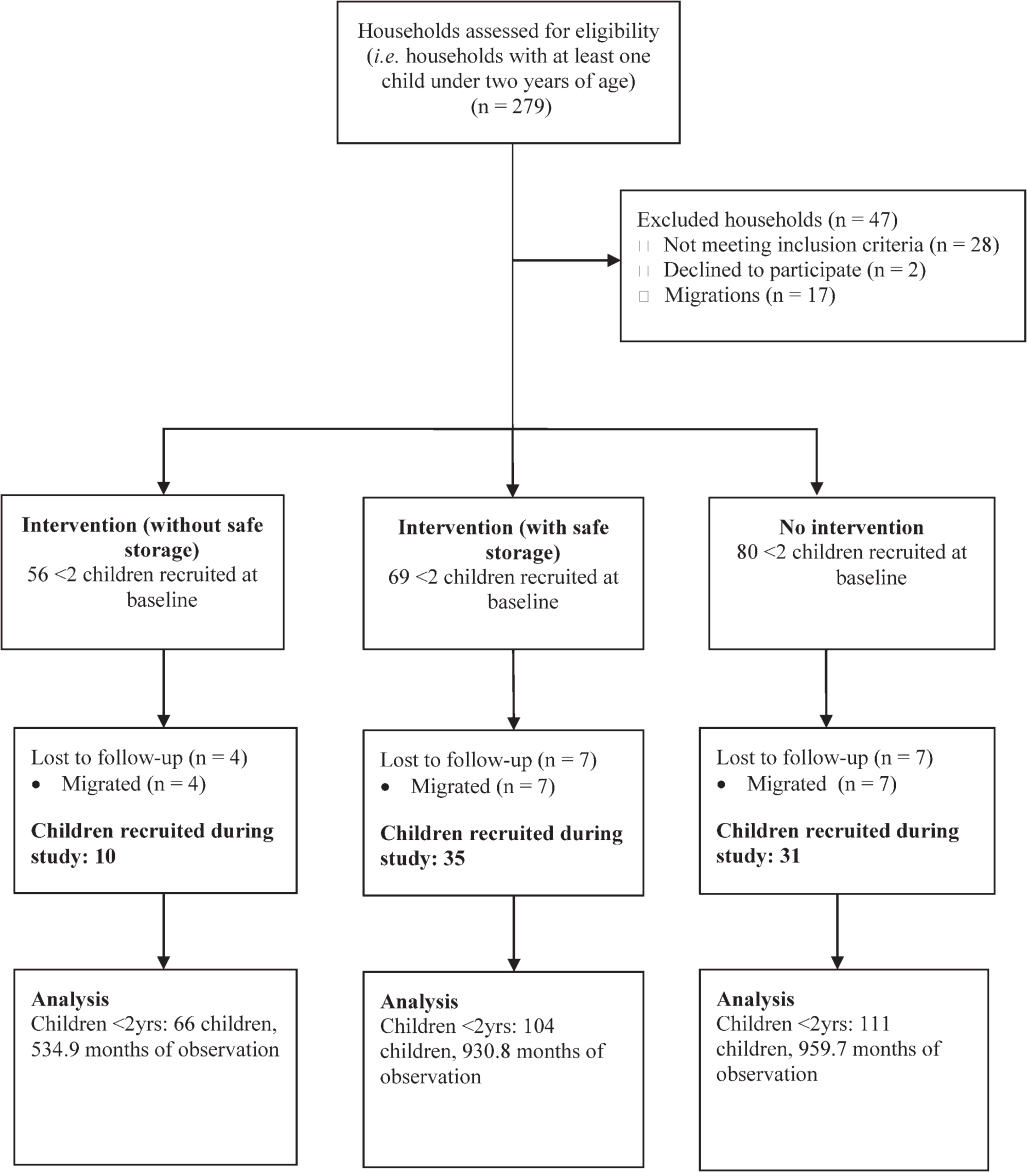
Consort diagram showing participant flow during the trial.

At baseline in October 2013, there were 279 households with at least one child under 2 years of age. Of the 232 eligible households, 205 consented to participate in the study, whereas 27 households felt the study activities would take up too much of their time and be an inconvenience to them (Figure 2

). The total number of children under the age of 2 years recruited until August 2014 was 281 with 111, 66, and 104 children in the no intervention, intervention without safe storage, and intervention with safe storage container, respectively. They contributed to a total of 203.2 child years of follow-up: 84.7% of the total anticipated follow-up time and average follow-up contributions of 0.73, 0.67, and 0.74 years per child for the no intervention, intervention without safe storage, and intervention villages with safe storage container, respectively.

### Baseline characteristics.

A majority (71.6%) of the study families belonged to the lower socioeconomic strata. The primary drinking water sources were public taps (54.2%), followed by private wells (21.3%), and house tap connections (16.9%) (Table 1). A minority (12.8%) of the families reported treating water regularly at home, mainly by boiling. Nearly 94% of families reported washing hands with only water after defecation. Only one-third (91/268, 33.3%) of the families reported having a toilet and of them, 81.3% (74/91) reported using the toilets on a regular basis. Significant differences were observed between the study villages for mean number of individuals and children per household, type of family, primary source of drinking water, water treatment, toileting, and waste-disposal practices (Table 1).

### Water quality.

There were 167 source-water, 60 paired (pre/postfiltration) membrane-filter, and 177 household-container water samples (total 464 samples) collected during the study. There was no residual chlorine in any of the source drinking water samples. Of source drinking water samples, 74% (149/202), 71.5% (128/179), and 67.3% (136/202) had detectable TC, FC, and *E. coli*, respectively. The intervention village without safe storage had the highest *E. coli* levels with an arithmetic mean (95% CI) of 92.4 (88.6–96.3) CFUs/100 mL followed by the intervention with safe storage and no intervention village at 35.6 (33.6–37.6) and 20.7 (20.0–21.5), respectively (*P* value for trend < 0.001) (Supplemental Table 1).

Overall, 65% (39/60) of prefiltration samples tested had microbiological contamination (TC, FC, or *E. coli*), whereas only 3% (2/60) of postfiltration samples in the intervention villages were contaminated. Postfiltration contaminated samples had median (interquartile range, IQR) values of 11 (6–16) and 2 (0–4) for TC and *E. coli*, respectively, whereas prefiltration samples had median (IQR) values of 22 (7–37) and 11.5 (0–23). Arithmetic means of pre- and postfiltration samples tested are presented in Table 2. The membrane filters achieved a LRV of 0.86 (0.69–1.06), 1.14 (0.99–1.30), and 0.79 (0.67–0.94) for TC, FC, and *E. coli*, respectively, when only paired samples with microbiological contamination prefiltration were analyzed. There were no differences in pH, nitrates, residual chlorine, hardness, and TDS between pre- and postfiltration samples for the Skyhydrant filter (Supplemental Table 2). Households provided with safe storage containers had the lowest *E. coli* levels with an arithmetic mean of 13.5 (12.5–14.5) CFUs/100 mL compared with 48.6 (46.7–50.5) CFUs/100 mL and 63.4 (61.6–65.3) CFUs/100 mL) for household without the safe storage container and the no intervention villages (*P* value for trend < 0.001) (Table 3).

### Diarrheal burden in the study villages.

A total of 200 episodes of diarrhea were reported in 119 children from 117 households; seven episodes were reported from more than one child belonging to the same household. The overall diarrheal incidence in children under 2 years of age reported as episodes per child-year (95% CI) were 1.05 (0.85–1.30), 1.22 (0.94–1.59), and 0.79 (0.62–1.02) for the no intervention, intervention without safe storage, and intervention with safe storage villages, respectively. The number of children who ever had an episode of diarrhea did not significantly differ between the study villages. Diarrheal incidence appeared to peak between May and July, but was consistently lower for the intervention village with safe storage compared with the other two villages for all months of the study. The median (IQR) length of a diarrheal episode was 2 (2–3) days, similar for all the study villages. Diarrheal incidence was similar for male and female children in the study: 0.99 (0.81–1.21) and 0.98 (0.80–1.20) episodes per child-year, respectively.

In the univariate analysis, a 23% (IRR [95% CI] = 0.77 [0.52–1.13], *P* = 0.190) reduction in under-two diarrheal incidence rate was observed for the intervention with safe storage village versus the no intervention village and a 15% (IRR [95% CI] = 1.15 [0.75–1.77], *P* = 0.517) increase in under-two diarrheal incidence rate was observed for the intervention without safe storage village as compared with the no intervention village.

In the multivariate analysis, the reduction in diarrheal incidence for the intervention with safe storage compared with the no intervention village was 24% (IRR [95% CI] = 0.76 [0.51–1.13], *P* = 0.178) and the intervention village without safe storage had a 14% increase under-two diarrheal rates compared with no intervention (IRR [95% CI] = 1.14 [0.75–1.77], *P* = 0.530). However, none of the reported differences in diarrheal rates between study villages were statistically significant. Also, diarrheal rates did not appear to vary by gender and appeared highest for children 6–12 months of age (IRR [95% CI] = 1.30 [0.85–1.98], *P* = 0.222) compared with the referent category of children 0–6 months of age. The results of the multivariate analysis are presented in Table 4.

### Diarrhea and water quality.

We also explored the relationship between diarrheal incidence and average TC, FC, and *E. coli* contamination in household container samples in a subset of study households (*N* = 122). Increased diarrheal rates were observed with increasing levels of median microbiological contamination (TC, FC, and *E. coli*) in household container samples used independently in the survival regression models, although not statistically significant (Table 5).

The adjusted IRRs were (1.59 [0.78–3.26], *P* = 0.199) and (2.18 [090–5.28], *P* = 0.084) for household container samples with FC levels of 1–180 CFU/100 mL and > 180 CFU/100 mL, respectively, relative to the lowest contamination group of < 1 CFU/100 mL (Table 5).

## Discussion

The membrane filters produced microbiologically safe drinking water over the 1 year of testing with failures observed in only two of 60 postfiltration samples, possibly due to manual contamination of the storage tanks at the time of cleaning or water collection. The filters did not alter the physicochemical quality of water,^18^ and can be used to provide safe water to smaller communities, especially in places without a continuous power supply. This is also a potentially scalable intervention to meet the requirements of larger communities.^19^ The membrane filter achieved a log reduction of ∼1 for FC in field conditions, lower than what was reported from the laboratory assessment of a similar portable gravity-fed ultrafiltration device intended for household use,^25^ but it must be noted that LRVs are contingent upon the concentration of microorganisms in prefiltered water, and in this field study, only a small proportion of prefiltration water samples had high levels of contamination. The membrane filter might achieve higher LRVs, that is, higher elimination of indicator organisms in highly contaminated drinking water.

Tested as an alternative to available point-of-use methods of water filtration, decentralized membrane filtration with or without safe storage was not protective against childhood diarrhea. Most field trials evaluating the effect of water quality on diarrhea in India have tended to focus on point-of-use disinfection; and while methods such as household chlorination, gravity filters (such as ceramic filters) and boiling have shown potential for microbiological disinfection, low compliance and acceptability reduce the benefits consumers might gain from their use.^11^,^26^–^29^ Improving access to clean drinking water is millennium development goal 7c, and is the responsibility of the Ministry of Drinking Water and Sanitation in India.

The lack of association between safer water and childhood diarrhea in this study highlights the possible role of other modes of transmission that might drive endemic diarrhea among children in rural Vellore as reported by an earlier study.^30^ Another critical factor contributing to the observed lack of association could be reduced adherence to the study interventions in the intervention villages. Previous studies testing water quality interventions in the region have reported varying levels of adherence, especially for point-of-use methods of water disinfection.^11^,^13^ Adherence to the interventions was not assessed in our study, as it was intended to provide a more realistic estimate of the impact of decentralized membrane filtration if implemented as part of either governmental or nongovernmental programs in the region. High adherence to water quality interventions is crucial to realizing the health gains from them,^29^ and we have reason to believe that adherence in our study was high as the filtered drinking water provided at no cost was highly valuable to the families.^22^ Nevertheless, timely research on improving adherence and factors influencing acceptance to such water quality interventions is crucial to their long-term success in resource-limited settings.

This study had a few limitations: the intervention was provided at the community level, limiting its application to clusters (villages), and therefore, individual randomization could not be done. Since intervention allocation was purposive, the effect estimates in our study may be biased due to the presence of unmeasured confounding between the study villages. The villages selected for study were expected to have similar social and demographic characteristics, water sourcing and handling, and personal and household hygiene indicators. Baseline data collection therefore sought to exhaustively measure all known and potential confounders thought to differ between the study villages. The study villages were generally similar, except for observed differences in the numbers of individuals per household, family type, primary sources of drinking water, toileting, and waste-disposal practices (Table 1). Therefore, the effect of unmeasured confounding due to variables related to the measured baseline variables is expected to be negligible, if present. All study arms were provided with some form of intervention: the no intervention village was encouraged to continue the government-recommended central chlorination, and therefore, may not be an ideal “control” for the comparisons made with the intervention villages. Blinding of participants or study personnel was not possible; Cairncross and others report the difficulty in blinding such studies and urge caution.^31^ The primary outcome of study was diarrhea in children under 2 years of age, and a degree of underreporting of episodes may be likely, but we sought to reduce this by employing a twice-weekly surveillance using both home visit and telephonic interviews. Underreporting of diarrheal episodes may have still occurred in the no intervention village due to a lack of motivation among the families over time. We also failed to characterize the diarrheal episodes as having been reported during home visits or on telephonic surveillance. It is important to note that the consistently lower number of children in the intervention village without safe storage resulted in a lack of power to detect an effect on diarrhea. With the existing power, we would have been able to detect a difference of 27–31% between the intervention (without safe storage) and the no intervention. Also, the water quality of pretreatment samples may not accurately represent the water collected by study households as pretreatment samples were collected after flaming. Public taps have been implicated as a potential source of microbiological contamination in the past,^32^ and it is therefore likely that study households were exposed to more contaminated water than represented by the pretreatment samples. Moreover, the frequency and quantum of unfiltered water provided to children in the study could not be estimated. These data may have provided better estimates of household risk factors for diarrhea.

Microbiologically, the membrane filters were effective in improving the quality of water with detectable fecal contamination; however, the provision of membrane filtered water with or without safe storage containers was not protective against childhood diarrhea. Decentralized infrastructure for water filtration may be useful in regions where the microbiological quality of water is insufficiently addressed, provided initial costs for set up and maintenance are available.

## Supplementary Material

Supplemental Tables.

## Figures and Tables

**Table 1:** Baseline characteristics of study households (*N* = 273)

Characteristics	No intervention, *n* (%)	Intervention (without safe storage), *n* (%)	Intervention (with safe storage), *n* (%)	*P* value*
Recruitment details				
No. of households	101 (37.0)	67 (24.5)	105 (58.5)	–
Median (IQR) age at recruitment (months)	11.5 (5.6–17.0)	12.4 (7.2–18.0)	12.0 (6.0–16.4)	0.703
Demographic and socioeconomic				
Mean (SD) no. of individuals per household	6.1 (2.4)	5.3 (1.6)	5.6 (1.9)	0.032
Mean (SD) no. of children per household	1.9 (1.0)	1.7 (0.6)	1.7 (0.8)	0.043
Mean (SD) no. of rooms in the house	3.1 (1.9)	2.8 (1.3)	2.7 (1.4)	0.086
Socioeconomic status				
Lower	65 (66.3)	50 (74.6)	77 (74.8)	0.627
Middle	22 (22.5)	13 (19.4)	19 (18.5)	
Higher	11 (11.2)	4 (6.0)	7 (6.8)	
Type of house construction				
Pucca	79 (78.2)	52 (77.6)	82 (78.1)	0.485
Mixed	9 (8.9)	10 (14.9)	15 (14.3)	
Kutcha	13 (12.9)	5 (7.5)	8 (7.6)	
Type of family				
Joint	33 (32.7)	8 (11.9)	18 (17.1)	0.013
Extended	44 (43.6)	38 (56.7)	61 (58.1)	
Nuclear	24 (23.7)	21 (31.4)	26 (24.8)	
Utility bills per month	3.2 (0.9)	3.6 (1.0)	3.4 (0.9)	0.013
Primary drinking water source				
Public tap	44 (43.6)	29 (43.3)	75 (71.4)	< 0.001
Hand pump	1 (1.0)	0 (0.0)	0 (0.0)	
Rajiv Gandhi tank	1 (1.0)	8 (11.9)	4 (3.8)	
Private well	37 (36.6)	9 (13.4)	12 (11.4)	
Private borewell	3 (3.0)	2 (3.0)	2 (1.9)	
House tap	15 (14.8)	19 (28.4)	12 (11.5)	
Drinking water storage and treatment in the household				
Place of storage of drinking water container				
Inside the kitchen	87 (86.1)	54 (80.6)	81 (77.9)	0.247
Room apart from kitchen	14 (13.9)	13 (19.4)	23 (22.1)	
Treat water				
No	7 (6.9)	21 (31.3)	31 (29.5)	< 0.001
Occasionally	82 (81.2)	40 (59.7)	60 (57.1)	
Always	12 (11.9)	6 (9.0)	14 (13.4)	
Water treatment method†				
Filter with cloth	18 (17.8)	15 (22.4)	20 (19.1)	0.759
Boiling	91 (90.1)	42 (62.7)	64 (61.0)	< 0.001
Packaged water	1 (1.0)	0 (0.0)	4 (3.8)	–
Reverse osmosis or carbon filter	0 (0.0)	2 (3.0)	1 (1.0)	–
Personal and household hygiene				
Agent used to wash hands				
Only water	95 (94.1)	63 (94.0)	97 (92.0)	0.750
With soap and water	6 (5.9)	4 (6.0)	8 (8.0)	
Toileting				
Toilet present in house	27 (26.7)	31 (46.3)	33 (31.4)	0.023
Water present in toilet	27 (100.0)	31 (100.0)	33 (100.0)	–
Toilet used regularly	24 (88.9)	24 (77.4)	26 (78.8)	–
Household waste disposal†				
Within the compound	16 (15.8)	11 (16.4)	52 (49.5)	< 0.001
Outside the compound	85 (84.2)	50 (74.6)	48 (45.7)	< 0.001
Designated garbage bins	1 (1.0)	7 (10.5)	2 (1.9)	< 0.001
Burn waste	42 (41.6)	12 (17.9)	31 (29.5)	< 0.001

IQR = interquartile range; SD = standard deviation.

* *P* value for χ^2^ or analysis of variance F-test, Bonferroni-adjusted significance level: *P* < 0.003.

† Percentages do not add up to 100; individual responses compared for each category.

**Table 2:** Comparison of microbiological parameters in pre- and postfiltration samples for the membrane filters

Microbiological parameter (CFU/100 mL)	Arithmetic mean (95% CI)†		
	Prefiltration	Postfiltration	*P* value*
Unit 1			
Total coliforms	5.1 (3.9–6.5)	0 (0)	0.003
Fecal coliforms	7.8 (6.1–9.8)	0 (0)	0.005
*Escherichia coli*	4.3 (3.2–5.6)	0 (0)	0.003
Unit 2			
Total coliforms	5.9 (4.6–7.5)	0 (0)	< 0.001
Fecal coliforms	8.9 (7.0–11.1)	0 (0)	0.002
*E. coli*	3.0 (2.1–4.2)	0 (0)	0.003
Unit 3			
Total coliforms	36.0 (32.7–39.6)	1.3 (0.8–2.2)	0.004
Fecal coliforms	51.2 (46.7–56.1)	0 (0)	0.002
*E. coli*	27.0 (24.1–30.1)	0 (0)	0.006
Unit 4			
Total coliforms	13.2 (11.2–15.4)	0.5 (0.2–1.1)	< 0.001
Fecal coliforms	14.8 (12.4–17.5)	0 (0)	0.002
*E. coli*	8.9 (7.3–10.8)	0 (0)	< 0.001
Unit 5			
Total coliforms	12.4 (10.5–14.6)	0 (0)	0.001
Fecal coliforms	16.8 (14.2–19.7)	0 (0)	< 0.001
*E. coli*	9.3 (7.6–11.1)	0 (0)	< 0.001

CFU = colony-forming unit; CI = confidence interval.

* *P* value from paired *t* test.

† 95% CI calculated using Poisson distribution of microbiological parameters.

**Table 3:** Comparison of average TC, FC, and *Escherichia coli* at the point of use in the no intervention and intervention with and without safe storage samples for the period of study

Microbiological parameters (CFU/100 mL)	Arithmetic mean (95% CI)†			*P* value*
	No intervention (*N* = 72)	Intervention without safe storage (*N* = 54)	Intervention with safe storage (*N* = 51)	
TC	70.2 (68.3–72.2)	52.7 (50.8–54.7)	14.9 (13.8–15.6)	0.009
FC	74.2 (72.2–76.2)	49.5 (47.6–51.4)	16.9 (15.7–18.1)	0.008
*E. coli*	63.4 (61.6–65.3)	48.6 (46.7–50.5)	13.5 (12.5–14.5)	0.024

CFU = colony-forming unit; CI = confidence interval; FC = fecal coliforms; TC = total coliforms.

* *P* value from paired *t* test.

† 95% CI calculated using Poisson distribution of microbiological parameters.

**Table 4:** Diarrheal episodes, time at risk, and diarrheal incidence rates in children under 2 years of age

Category	No. of children	No. of diarrheal episodes	Total time at risk (years)	Diarrheal incidence (episodes/child year)	Unadjusted IRR (95% CI)	Adjusted IRR* (95% CI)	*P* value
Study arm							
No intervention	109	85	80.8	1.05 (0.85–1.30)	Ref	Ref	–
Intervention (without safe storage)	65	54	44.1	1.22 (0.94–1.60)	1.15 (0.75–1.77)	1.14 (0.75–1.77)	0.530
Intervention (with safe storage)	102	61	76.8	0.79 (0.62–1.02)	0.77 (0.52–1.14)	0.76 (0.51–1.13)	0.178
Gender							
Male	146	102	102.7	0.99 (0.82–1.20)	Ref	Ref	–
Female	130	98	99.0	0.98 (0.81–1.21)	1.00 (0.71–1.40)	1.05 (0.74–1.48)	0.790
Age of child (months)†							
0–6	94	37	40.9	0.90 (0.65–1.25)	Ref	Ref	–
6–12	62	67	52.4	1.28 (1.00–1.62)	1.32 (0.87–2.00)	1.30 (0.85–1.98)	0.222
12–18	58	44	59.1	0.75 (0.55–1.00)	0.75 (0.47–1.21)	0.74 (0.46–1.19)	0.221
18–24	43	52	48.8	1.06 (0.81–1.40)	1.09 (0.69–1.74)	1.07 (0.67–1.70)	0.783

CI = confidence interval; IRR = incidence rate ratio.

* Adjusted for study arm, gender, social class, type of habitation, and hand-washing agent.

† Number of children contributing time at each age stratum.

**Table 5:** Effect of average water quality in the household on under-two diarrheal incidence (*N* = 122)

Category*	No. of children	No. of diarrheal episodes	Total time at risk (years)	Diarrheal incidence (per child-year)	Unadjusted IRR (95% CI)	Adjusted IRR (95% CI)†	*P* value
Total coliforms							
< 1 CFU/100 mL	21	16	18.1	0.88 (0.54–1.44)	Ref	Ref	–
1–180 CFU/100 mL	87	95	79.2	1.20 (0.98–1.47)	1.37 (0.73–2.57)	1.35 (0.67–2.73)	0.396
> 180 CFU/100 mL	14	13	11.1	1.17 (0.68–2.02)	1.38 (0.58–3.34)	1.66 (0.65–4.27)	0.292
Fecal coliforms							
< 1 CFU/100 mL	21	13	17.9	0.73 (0.42–1.25)	Ref	Ref	–
1–180 CFU/100 mL	83	93	76.9	1.21 (0.99–1.48)	1.67 (0.86–3.26)	1.59 (0.78–3.26)	0.199
> 180 CFU/100 mL	18	18	13.6	1.32 (0.83–2.10)	1.90 (0.82–4.40)	2.18 (0.90–5.28)	0.084
*E. coli*							
< 1 CFU/100 mL	28	25	24.4	1.03 (0.70–1.52)	Ref	Ref	–
1–180 CFU/100 mL	80	86	72.9	1.18 (0.95–1.46)	1.15 (0.67–1.98)	1.06 (0.58–1.97)	0.841
> 180 CFU/100 mL	14	13	11.1	1.17 (0.68–2.02)	1.19 (0.53–2.70)	1.34 (0.57–3.16)	0.504

CFU = colony-forming unit; CI = confidence interval; IRR = incidence rate ratio.

* Median values per household.

† Adjusted for study arm, gender, social class, type of habitation, and hand-washing agent.
