# Supplementary material for: Effectiveness of Membrane Filtration to Improve Drinking Water: A Quasi-Experimental Study from Rural Southern India
Source: Am J Trop Med Hyg. 2016 Nov 2;95(5):1192–200. doi: 10.4269/ajtmh.15-0675 (PMC5094238; doi:10.4269/ajtmh.15-0675)
Supplement: Supplementary file 1 [file SD9.pdf]

Supplemental Table 1

Comparison of arithmetic means of TC, FC, and *E. coli* in water sources of the study villages

Microbiological parameters (CFU/100 mL)	Arithmetic mean (95% CI)†			
	No intervention (*N* = 142)	Intervention without safe storage (*N* = 24)	Intervention with safe storage (*N* = 36)	*P* value*
TC	25.6 (24.8–26.5)	139.1 (134.4–143.9)	42.3 (40.2–44.5)	< 0.001
FC	24.6 (23.8–25.5)	100.5 (96.1–104.9)	43.2 (40.8–45.7)	< 0.001
E. coli	20.7 (20.0–21.5)	92.4 (88.6–96.3)	35.6 (33.6–37.6)	< 0.001

CFU = colony-forming unit; CI = confidence interval; FC = fecal coliforms; TC = total coliforms.
**P* value from analysis of variance F-test for global comparison between study arms.
†95% CI calculated using Poisson distribution of microbiological parameters.

Supplemental Table 2

Comparison of pre- and postfiltration values of physical parameters for the membrane filters

Parameter	Arithmetic mean (95% CI)		
	Prefiltration	Postfiltration	*P* value*
Unit 1			
pH	8.5 (8.3–8.7)	8.4 (8.2–8.6)	0.096
Residual chlorine	0 (0)	0 (0)	0.830
Nitrate	5.6 (1.5–9.6)	3.3 (0.5–7.2)	0.095
Hardness	500 (400–599)	458 (410–504)	0.123
TDS	1,364 (1,206–1,522)	1,381 (1,233–1,528)	0.715
Unit 2			
pH	8.3 (8.0–8.6)	8.3 (8.1–8.6)	0.539
Residual chlorine	0.1 (0.2–0.5)	0 (0)	0.169
Nitrate	11.7 (1.5–21.8)	9.4 (1.5–10.4)	0.173
Hardness	480 (345–615)	483 (398–567)	0.523
TDS	1,692 (1,612–1,773)	1,661 (1,550–1,771)	0.387
Unit 3			
pH	8.6 (8.3–8.8)	8.5 (8.2–8.8)	0.169
Residual chlorine	0 (0)	0 (0)	0.831
Nitrate	2.2 (1.2–5.6)	1.1 (1.1–3.7)	0.173
Hardness	415 (306–524)	418 (318–517)	0.551
TDS	893 (867–919)	892 (860–923)	0.456
Unit 4			
pH	8.2 (7.8–8.5)	8.2 (7.8–8.5)	0.380
Residual chlorine	0 (0)	0 (0)	–
Nitrate	21.7 (8.2–35.1)	22.8 (1.5–47.0)	0.555
Hardness	574 (519–630)	579 (497–660)	0.556
TDS	1,600 (1,401–1,799)	1,530 (1,371–1,690)	0.269
Unit 5			
pH	8.3 (8.0–8.6)	8.3 (8.0–8.6)	0.410
Residual chlorine	0 (0)	0 (0)	–
Nitrate	1.1 (1.1–3.7)	1.1 (1.1–3.7)	0.500
Hardness	567 (506–629)	529 (467–582)	0.023
TDS	1,490 (1,414–1,566)	1,505 (1,441–1,570)	0.757

CI = confidence interval; TDS = total dissolved solids.
*Comparisons using paired *t* test.
